# Technical factors can impact on remote consultations in rheumatology: results from a service evaluation during the COVID-19 pandemic

**DOI:** 10.1007/s00296-022-05112-5

**Published:** 2022-04-11

**Authors:** Sreekanth Vasireddy, Surabhi Wig, Michael Hannides

**Affiliations:** 1 Department of Rheumatology, Bolton One Health Centre, Bolton NHS FT, Moor Lane, Bolton, BL3 5BN UK; 2 School of Biological Sciences, University of Manchester, Manchester, UK

**Keywords:** Remote consultations, Video consultations, Rheumatology clinics, Technical factors, COVID-19, Management plan

## Abstract

Since the COVID-19 pandemic started, there have been changes in clinical practice to limit transmission, such as switching from face-to-face to remote consultations. We aimed to study the influence of technical factors on remote consultations in our experience during the pandemic. 12 clinicians completed data collection forms after consultations, recording the technology used (video vs phone); technical problems encountered; discharge or subsequent appointment status; and technical aspects of the consultation process using 0–10 numerical rating scales (NRS) (Time Adequate; Relevant History; Physical Exam; Management Plan; and Communication Quality). Data were collated on an MS Access 2016 database and transferred to SPSS version 25 for statistics. Of 285 forms valid for analysis, 48 (16.8%) had video consultations. Of 259 forms with technical problems data recorded, 48 (18.5%) had a technical problem. Video patients were significantly younger (mean 49.3 vs 61.3 years, *p* < 0.001), had higher scores on Physical Exam scale (mean 4.0 vs 2.6, *p* < 0.001), but had no significant difference on Management Plan scale (7.3 vs 7.2). Those with technical problems were more common among video consultations (33.3% vs 15.4%, *p* = 0.005), had lower scores on Time Adequate scale (7.7 vs 8.7, *p* < 0.001) and Communication Quality scale (7.1 vs 8.4, *p* < 0.001), but had no significant difference on Management Plan scale (7.3 vs 7.2). The strongest correlation of Management Plan scale was with Communication Quality scale (Rho = 0.64). Of the NRS, a 1-point reduction in scores on Management Plan scale was the strongest predictor of subsequent face-to-face appointment (Odds Ratio 1.88, 95% CI 1.58–2.24), and this remained an independent predictor in multivariate analysis (adjusted OR 1.90, 1.57–2.31). Having a technical problem was inversely associated with the outcome of a subsequent face-to-face appointment (OR 0.17, 0.04–0.74), and this remained significant after adjustment for Management Plan in multivariate analysis (adjusted OR 0.09, 0.12–0.54). Video patients were younger suggesting a preference for video amongst younger patients. Although technical problems were more common with video, having a video consultation or a technical problem had no significant impact on management plan. Scoring lower on the Management Plan scale was the strongest predictor of, and independently associated with, requesting a subsequent face-to-face appointment. The inverse relationship of technical problems with subsequent face-to-face appointment request will need validation in further studies.

**Supplementary Information:**

The online version contains supplementary material available at 10.1007/s00296-022-05112-5.

## Introduction

The coronavirus 19 disease (COVID-19) pandemic has changed clinical practice in various specialties toward limiting transmission and reducing severity of infections. Patients with rheumatic disease are no more likely, and probably less likely, to get infected compared to the general population, probably because of better health protection behaviours from prior education regarding general infection risk [1]. However, once infected, patients with underlying chronic health problems have been shown to have a worse prognosis [2]. Many rheumatic conditions and treatments in rheumatology could be associated with lowered immunity and potential worse prognosis with infection, including COVID-19 [3]. As the pandemic took hold in the UK, guidance was issued by National Health Service England (NHSE), National Institute for Health and Care Excellence (NICE) and the British Society for Rheumatology for managing rheumatology patients during the pandemic [4–6]. This included guidance regarding moving from face-to-face consultations to remote appointments delivered through telephone and video.

Remote consultations are a part of telemedicine which can be loosely defined as the use of communications technology for the provision of health care [7]. In this sense, the concept was noted as early as 1878 when clinical assessment of cardiac sounds transmitted for many miles using a microphone and telephone was conceived [8]. Telephone consultation was also reported as early as 1928 in a study of eclampsia as being helpful when bedside management is not possible [9]. Since then, there has been increasing uptake of aspects of telemedicine in various specialities including rheumatology, although evidence of its effectiveness for the diagnosis and management of rheumatic disease was recently found to be limited [7].

Positive experiences have been published in running nurse-led telephone follow-up clinics for rheumatic disease patients in the pre-COVID era [10]. Similarly, nurse-led telephone follow-up clinics have been found to be effective in monitoring parenteral osteoporosis treatment [11]. Studies have also been done comparing telephone versus video consultations with respect to efficacy and associated technical problems [12, 13]. However, there have been no published studies commenting on the technical aspects, or tools for assessing the technical aspects of the consultation process itself during remote or indeed face-to-face consultations.

During the pandemic, video consultation facility was made more widely available in the UK [14]. In our department, we switched all routine outpatient appointments to remote consultations following COVID-19 guidance. In the end of March and April 2020, all appointments were offered as phone consultations. In May, laptop computers with video call software were procured for video consultations. The process of switching from face-to-face to remote consultations was implemented using a quality improvement approach with a view to evaluating the service and deriving any lessons for future enhancements. In this analysis from the service evaluation, we aimed to look at how various technical factors impacted on the efficacy and outcomes of remote consultations used for an unselected case mix of non-urgent rheumatology patients.

## Methods

Practical issues for implementing remote consultations were discussed in multi-disciplinary team meetings and refined over the initial months until a stable pattern of delivery was achieved. The wider initiative was then registered as a quality improvement project with the Clinical Effectiveness Department in our Trust before data collection (Safeguard registration no.: 3718).

### Outpatient department

During the pandemic, we continued to deliver clinics from the usual dedicated clinic rooms in our rheumatology outpatient department suite. Clinic rooms had pre-installed Cisco^®^ CP 7821 digital landline phones with high-fidelity corded handsets and hands-free speakers for phone consultations. High-quality Wi-fi signal was available in clinic rooms for video consultations via Lenovo^®^ V130-14IKB laptops with NHS Attend Anywhere^®^ video call software.

### Data collection form

A data collection form was developed incorporating items regarding the technology used (video vs phone), technical problems encountered, discharge status and the status of subsequent appointment requested by the clinician (face-to-face or remote). For technical problems, six checkboxes were available with the option of checking one or more boxes as described: no issues; poor video quality; poor audio quality; poor connection (e.g. delay, re-connection, disconnection, call failure, etc.); capability of user; and ‘other’ with space for free text.

To assess the technical aspects of the consultation process itself, 11-point (0–10) numerical rating scales (NRS) were designed and incorporated into the form in a questionnaire format: adequate time for the consultation (Time Adequate scale); relevant history achieved (Relevant History scale); physical examination findings obtained (Physical Exam scale); management plan achieved (Management Plan scale); and overall quality of the communication (Communication Quality scale). Clinicians were asked to score the effectiveness on the scales compared to what could be expected at a face-to-face appointment (NRS with definitions of 0 and 10 endpoints used in data form are shown in Online Resource 1).

### Patients

All non-urgent new and follow-up appointments were converted to remote appointments. Patients were offered the choice of either video or phone consultation by the booking team at the time of booking the appointment, except for pharmacist clinics where phone consultations were offered routinely unless patient requested video consultation (as our Pharmacists do not do physical examination). At the end of consultation, plans for discharge or subsequent appointment (face-to-face or remote consultation) were discussed and agreed with the patient.

Inclusion criteria: All remote consultations during the data collection period (unselected rheumatology case mix).

Exclusion criteria: All face-to-face consultations during the data collection period.

### Clinicians

Clinicians who regularly attended clinics in the department completed data collection forms. This included 3 consultant rheumatologists, 2 senior rheumatology trainees (speciality training registrars), 1 foundation year-2 doctor, 3 specialist nurses, 1 advanced rheumatology practitioner and 2 senior rheumatology pharmacists (total = 12). Before starting the project, a PowerPoint presentation was delivered to participating clinicians describing the project.

### Data collection and statistical analysis

Data were collected between 15/10/2020 and 09/11/2020. Individual case notes were prepared with data collection forms and clinicians completed forms after each appointment. Forms were collected at the end of each clinic and screened to ensure completeness. Data were collated on a Microsoft Access 2016 database. Data excluding patient identifiers were transferred to SPSS version 25 for statistical analysis. Variables were processed to derive dichotomous groups where appropriate. For all comparisons and associations *p* < 0.05 was considered significant.

*Comparisons of means*: For age and the NRS, comparisons of means were performed between dichotomous groups and expressed with standard deviation. Significance of any differences was assessed by Mann–Whitney *U* test.

*Comparisons of proportions*: Proportional distributions between dichotomous groups were expressed as percentages. Significance of any differences was assessed using Chi-square tests.

*Associations among NRS*: Relationships between the NRS were studied using Spearman correlation coefficient (Rho). Correlations were classified as “strong” if Rho was > 0.6; “moderate” if Rho was 0.4–0.6; and “weak” if Rho was 0.2–0.4.

*Odds ratios:* For assessing strength of association of the variables with subsequent face-to-face appointment status as dependent variable, univariate logistic regression was used. Multivariate logistic regression models were used to ascertain independent predictors (both expressed as odds ratios and 95% confidence intervals).

## Results

### Descriptive statistics

During the study period, 324 (100%) forms were returned from 324 booked remote appointments from the clinics. Of these, 39 were excluded (did not attend = 19 (5.9%); cancelled appointment = 6 (1.9%); no patient identifier/not possible to validate = 14 (4.3%)). The remaining 285 (88%) valid forms were entered on to the database (Table 1), comprising 284 separate patients (1 patient had 2 consultations within the period with different clinicians). Of these, 193 (67.7%) were women. Total new appointments were 51 (17.9%). 48 (16.8%) had video consultations. Data were missing regarding technical problems in 26 forms. Of the remaining 259 forms, 48 (18.5%) recorded experiencing at least one technical problem. No clinician had significant vision or hearing impairment affecting consultation processes. Of the technical problems recorded, one was attributable to the provider site (clinician had to switch laptop as no video access with initial laptop); the rest were attributable to patients (additional data on types and frequencies of technical problems are given in Online Resource 2).

**Table 1:** Descriptive statistics of the whole cohort (*n* = 285)

Variables	Value
Mean age (min–max) (years)	59.3 (19.3–89.2)
Gender (female: male)	193: 92
Consultation method (video vs telephone)	48 vs 237
Technical problems	
Not recorded	26
No problems	211
Problems reported	48
Appointment outcome	
Not recorded	12
Discharged	24
Reappointment requested	249
Face to face	55
Remote (video or telephone)	194
Type not recorded	36

Subsequent appointment status information was missing in 12 forms. Of the remaining 273, 24 (8.8%) patients were discharged. Of the 249 who were offered a subsequent appointment, 55 (22.1%) were offered a face-to-face consultation. Across the whole cohort, the NRS reflected a full range of scoring, with a mean score of 1.6 for Physical Exam scale, but other scales had mean scores of over 7 (Table 2). As data for age and the NRS were not normally distributed, non-parametric tests were applied for further analysis (additional data on distribution and tests of normality are given in Online Resource 3).

**Table 2:** Performance of numerical rating scales (NRS, 0–10) across the whole cohort (*n* = 285)

NRS	Number recorded	Mean	Median	Minimum scored	Maximum scored
Time adequate	284	8.50	9	0	10
Relevant History	283	8.75	9	2	10
Physical examination	240	1.60	0	0	10
Management plan	283	7.22	8	0	10
Communication quality	283	8.11	9	2	10

### Video vs phone consultation

*Age:* Video consultations patients were significantly younger (mean 49.3 ± SD 16.1 vs 61.3 ± 17.6 years, *p* < 0.001).

*Patient gender:* 34 (17.6%) women and 14 (15.2%) men were listed for a video consultation and the rest for phone consultation (*p* = 0.61).

*New vs follow-up:* 10 (19.6%) of the new patients and 38 (16.2%) of the follow-up patients were listed for video consultation with the rest listed for phone consultation (*p* = 0.56).

**Fig. 1:**
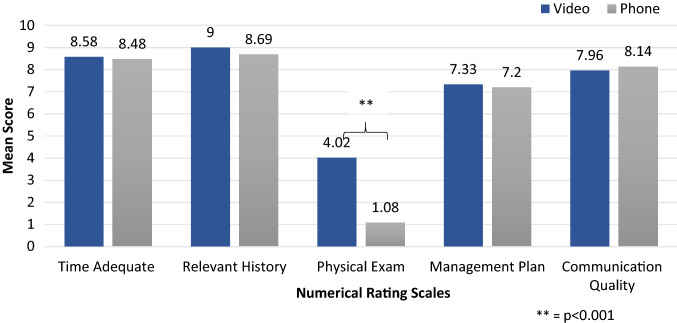
Mean scores on numerical rating scales in video vs phone consultations

*Numerical rating scales (Fig. **1**):* Video consultations were scored higher on the Physical Exam scale (4.0 ± 3.7 vs 1.1 ± 2.6, *p* < 0.001). There were no significant differences between the video and phone consultation scores for Time Adequate scale (8.6 ± 1.5 vs 8.5 ± 1.6); Relevant History scale (9.0 ± 1.1 vs 8.7 ± 1.2); Management Plan scale (7.3 ± 2.6 vs 7.2 ± 2.4); and Communication Quality scale (8.0 ± 2.1 vs 8.1 ± 1.9).

*Subsequent appointment status:* There was no significant difference between video and phone groups with respect to discharge status (*n* = 3, 7% vs *n* = 21, 9.1%, *p* = 0.65). Of those offered subsequent appointments, there was a non-significant trend for patients who had video consultations to be listed for subsequent face-to-face appointments (*n* = 13, 32.5% vs *n* = 42, 20.1% respectively; *p* = 0.08).

### Technical problems

*Age:* There was no significant difference in age between consultations with technical problems and those with no technical problems (60.3 ± 18.1 vs 59.4 ± 18.0, *p* = 0.73).

*Patient gender:* 31 women (17.8%) and 17 men (20%) were recorded as having a technical problem (*p* = 0.67).

*New vs follow-up:* 8 (17%) of new patients and 40 (18.9%) of follow-up patients were recorded as having a technical problem (*p* = 0.77).

*Technical problems and Video vs phone:* A significantly higher proportion of video consultations (*n* = 15, 33.3%) had technical problems compared to phone consultations (*n* = 33, 15.4%; *p* = 0.005).

**Fig. 2:**
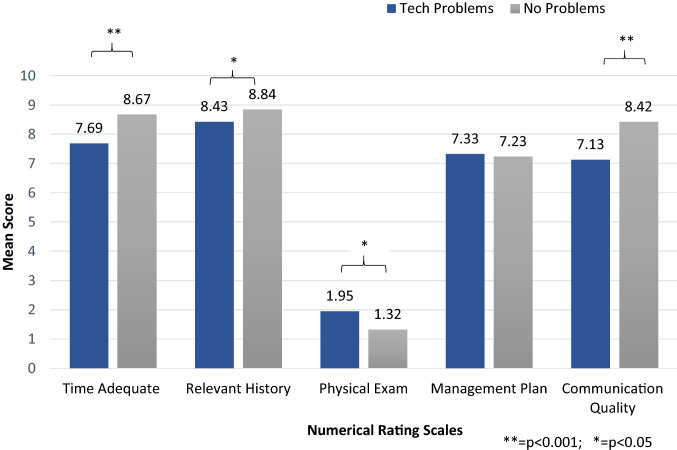
Mean scores on numerical rating scales in consultations with technical problems vs no problems

*Numerical rating scales (Fig. **2**):* Those with technical problems had lower mean scores on the Time Adequate scale (7.7 ± 2.1 vs 8.7 ± 1.5, *p* < 0.001); Relevant History scale (8.43 ± 1.1 vs 8.84 ± 1.2, *p* = 0.007); Communication Quality scale (7.1 ± 1.8 vs 8.4 ± 1.6, *p* < 0.001). There was no significant difference in the Management Plan scale (7.3 ± 2.1 vs 7.2 ± 2.5). Although both groups had low mean scores on the Physical Exam scale, those with technical problems scored slightly better (2.0 ± 3.0 vs 1.3 ± 2.8, *p* = 0.023). This is probably because there was a higher proportion of video consultations in the technical problems cohort (video patients had a higher mean score for Physical Exam scale as noted earlier). After adjusting for video consultation in a multivariate logistic regression model, Physical Exam scale was not significantly associated with technical problems (*p* = 0.67).

*Subsequent appointment status:* A similar proportion of those with technical problems (*n* = 4, 9.5%) and with no technical problems (*n* = 19, 9.2%) were discharged (*p* = 0.95). Interestingly, a significantly smaller proportion of patients with technical problems were offered subsequent face-to-face appointment (*n* = 2, 5.3%) compared to those with no technical problems (*n* = 46, 24.6%; *p* = 0.008).

### Correlations among numerical rating scales

There were no negative correlations between any of the NRS. There were positive correlations amongst the scales ranging from strong to weak.

*Strong correlations (all p* < *0.001):* Strong correlations were noted between the Relevant History scale and Communication Quality scale (correlation coefficient Rho = 0.74); Relevant History scale and Time Adequate scale (Rho = 0.65); Management Plan scale and Communication Quality scale (Rho = 0.64); and Management Plan scale and Relevant History scale (Rho = 0.63) (Table 3).

**Table 3:** Correlations of Management Plan scale with other numerical rating scales (NRS): Spearman correlation coefficient (Rho) (all *p* ˂0.001)

NRS	Correlation with Management Plan scale (Rho)
Communication Quality scale	0.64
Relevant History scale	0.63
Time Adequate scale	0.44
Physical exam scale	0.28

*Moderate correlations (all p* < *0.001):* Moderate strength correlations were noted between Communication Quality scale and Time Adequate scale (Rho = 0.51); and Management Plan scale and Time Adequate scale (Rho = 0.44) (Table 3).

*Weak correlations (p* ≤ *0.001):* Weak positive correlations were noted between Physical Exam scale and Relevant History scale (Rho = 0.33); Physical Exam scale and Management Plan scale (Rho = 0.28) (Table 3); Physical Exam scale and Time Adequate scale (Rho = 0.24); and Physical Exam scale and Communication Quality scale (Rho = 0.22).

### Numerical rating scales and subsequent appointment status

*Discharge vs re-appointment:* There were no significant differences in the mean scores between discharged patients and those offered a subsequent appointment on Time Adequate scale (8.5 ± 1.9 vs 8.5 ± 1.6); Relevant History scale (8.6 ± 1.0 vs 8.7 ± 1.3); Physical Exam scale (1.4 ± 3.2 vs 1.5 ± 2.9); Management Plan scale (8.1 ± 1.3 vs 7.1 ± 2.5); and Communication Quality scale (8.2 ± 1.2 vs 8.1 ± 1.9).

*Face-to-face vs remote re-appointment:* Those offered subsequent face-to-face appointments had lower mean scores for Time Adequate scale (8.2 ± 1.4 vs 8.6 ± 1.6, *p* = 0.008), Relevant History scale (8.2 ± 1.4 vs 8.9 ± 1.2, *p* = 0.001); Management Plan scale (4.5 ± 2.7 vs 7.9 ± 1.8, *p* < 0.001); and Communication Quality scale (6.5 ± 2.2 vs 8.6 ± 1.5, *p* < 0.001). There was a non-significant trend for lower mean score in the Physical Exam scale also for face-to-face appointments group (1.2 ± 2.5 vs 1.6 ± 3.1, *p* = 0.25).

### Odd ratios (subsequent face-to-face appointment request)

The NRS were put through univariate logistic regression to assess strength of association with the likelihood of subsequent face-to-face appointments (dependent variable) with each 1-point reduction in the scores. The Time Adequate scale was not significantly associated with an odds ratio (OR) of 1.14 (95% confidence intervals (CI) 0.96–1.35, *p* = 0.149). Relevant History scale was significantly associated with OR 1.47 (95% CI 1.16–1.86, *p* = 0.002). Management Plan scale had the strongest association with OR 1.88 (95% CI 1.58–2.24, *p*˂0.001). Communication Quality scale also had a significant association with OR 1.77 (95% CI 1.48–2.11, *p*˂0.001).

Technical problems were inversely associated with subsequent face-to-face appointment with OR 0.17 (95% CI 0.04–0.74, *p* = 0.018). After adjusting for video consultations and Physical Exam scores in a multivariate logistic regression model, technical problems remained inversely associated with subsequent face-to-face appointments (dependent variable) (adjusted OR 0.18, 95% CI 0.04–0.79, *p* = 0.023). In a further multivariate regression model, both technical problems (adjusted OR 0.09, 95% CI 0.12–0.54, *p* = 0.009) and a 1-point reduction in Management Plan scale (adjusted OR 1.90, 95% CI 1.57–2.31, *p*˂0.001) remained independent predictors of a subsequent face-to-face appointment request.

## Discussion

The move towards delivering remote consultations was important to reduce the risk of COVID-19 incidence in immune-compromised patients, and also for non-urgent care to be continued to be delivered effectively over sustained periods. We implemented our switch to remote consultations using a quality improvement approach with a view to studying the impact of various factors on the delivery of these consultations in an unselected case mix of rheumatology patients. In this study, we did not collect specific diagnosis or treatment data so that the analysis focused on factors that could potentially be generalisable to other areas of clinical practice.

### Video consultations

Video consultations formed a smaller proportion compared to phone consultations in our analysis. Although younger patients seem to have taken up the video option, there was no significant difference in the uptake between men and women and indeed new or follow-up status. This is in keeping with the finding that video enabled telemedicine usage was highest in the 20–44 years age group in a recent study [15]. This is also similar to the finding that video consultation participants were younger compared to both phone consultation and face-to-face consultation participants in a general practice study in the pre-COVID era [13]. In the same study, the proportion of female participants was similar in video and phone consultations (54% vs 55%) although this was smaller for face-to-face consultations (39%) [13]. The higher proportion of phone consultations is also in keeping with an international survey of patients which found telephone being the predominant modality in Canada (77%) and other countries (83.3%), although in the USA, this was 44.1% [16]. In our study, video consultations scored higher for physical examination although the mean score was quite low (4.0 out of a maximum of 10). Despite these higher scores there was no significant difference between the video and phone consultation with respect to achieving a management plan or the outcome of discharge or subsequent follow-up appointments. This lack of impact of the physical examination in the video consultation on the management plan could be in keeping with a previous finding that, where it was important, clinical signs could not be visualised over the video and that there was no significant difference in the diagnostic accuracy between video and telephone communication [12]. A subsequent study by the same group using a desktop video conferencing unit and a conventional video camera with zoom lens for close up picture reported that diagnostic accuracy was higher with video consultation (97%) compared to telephone consultation (71%) against a gold standard confirmation by face-to-face consultation [17]. This suggests that higher quality image capture, and the format of a GP-facilitated consultation with a specialist as in this study, can be associated with better diagnostic accuracy. However, in our pragmatic clinical setting, patients used mobile phones, tablet devices, laptops/desktops with variable quality and resolution of in-built cameras for image capture, and in uncontrolled domestic or work settings, which could have had an impact on the information gathered.

### Technical problems

In our analysis, a greater proportion of video consultations had technical problems compared to phone consultations. The potential for technical problems confounding remote consultations was flagged up as early as the conceiving of telemedicine itself when it was observed in 1878 that a microphone could be used to capture cardiac sounds and transmitted over a telephone line, but with a cautionary note that there is a limit to the capabilities of the microphone [8]. More recently, technical problems were also found to be common and it was felt infrastructure issues need to be addressed before video consultations can be mainstream in primary care [13]. In a survey of clinicians, it was found that video consultations were less frequently used than telephone consultation even when video consultation facility was available since the onset of the COVID-19 pandemic [14]. The perception and potential for more frequent technical problems with video consultations could be a reason for this. Similarly, in a survey of clinicians from the Netherlands, 99% of the respondents used telephone and 9% of them used video for providing consultations for continuity of care [18]. Irrespective of the mode of consultation, the occurrence of a technical problem did not have a significant impact on the management plan itself in our study and this is reassuring. Indeed, in this analysis, the patients who had a technical problem seemed to score slightly better on the Physical Exam scale but this could be simply a reflection that the technical problems were more common in the video consultations which, in turn, scored better on the Physical Exam scale. Interestingly, the occurrence of a technical problem did not seem to lead to a subsequent face-to-face appointment request, and indeed, there was an inverse relationship, independent of other variables. In view of the small numbers, the clinical significance of this finding is uncertain, but other factors that we did not study could potentially explain the finding, for example underlying clinical diagnosis etc. This finding would need to be further explored in larger studies in the future for validation and understanding the clinical relevance if any.

### Numerical rating scales

A 0–10 scale was used for rating the quality of care delivery in a survey of clinicians from the Netherlands in the early months of COVID-19 pandemic, where the average score of 7 was reported [18]. However, on a literature search, we did not find any tools including scales published that were validated for evaluating the technical aspects of clinical consultations, including remote consultations. We therefore designed NRS to assess the technical elements of a clinical consultation without reference to the underlying condition to get a broad understanding of the technical factors impacting on the consultation and outcome processes independent of the underlying clinical condition and its severity. As this was a pragmatic service evaluation under a Quality Improvement project design, we did not separately validate these NRS scales. However, the NRS we used have all had significant positive correlations amongst themselves suggesting that this design is a valid tool for such an assessment. Similarly, the scales seem to have performed well to differentiate between the dichotomous variables studied, including association also with subsequent appointment status, suggesting that they are valid tools for such an enquiry. The Management Plan scale had the strongest association with subsequent face-to-face appointment and was an independent predictor after adjusting for other significant dichotomous variables. The Management Plan scale correlated strongly with Communication Quality scale and Relevant History scale and only had a moderate correlation with Time Adequate scale and Physical Exam scale. Interestingly, Relevant History scale strongly correlated with overall Communication Quality scale as well as Time Adequate scale. This suggests that while adequate time may have a strong association with relevant history, the obtaining of relevant history of itself seems to be more directly and strongly related to the management plan rather than it being simply a result of having adequate time of itself. This was also borne out by the finding that although time was found to be less adequate when there was a technical problem, this did not impact on the management plan scores in this analysis. In a previous study, video consultations were found to be slightly longer than telephone consultations but shorter than face-to-face consultations, and overall consultation quality was considered very similar between telephone and video, although no report was made on management plan in this study [13].

### Discharge status

Although numbers were small, it was reassuring to note that there were no significant differences in discharge rates between video and phone consultations, or between those with technical problems and without technical problems. It is also reassuring that there were no significant differences in the NRS between those discharged and not discharged, suggesting that the discharge decision is likely to have been made based mainly on the underlying clinical condition (8.8% overall discharged). This rate is in keeping with recently published discharge rate from tele-clinics of 10% [19].

### Limitations

This analysis is from a service evaluation designed ultimately to improve quality in our service, and the results potentially may not be generalizable to other service settings. However, the general themes brought out here may be useful in designing evaluations and improvements in other service settings. Patient-related factors and clinician-related factors may also have independent effects on remote consultations, but due to the volume of data involved, these are the subjects of two further analyses planned for the future from this study. As our objective was to study remote consultations in an unselected case-mix of non-urgent rheumatology patients in a pragmatic out-patient clinic setting, we did not collect information regarding the patients’ diagnoses, severity of condition or ongoing management for this analysis. It is possible that some of the outcomes such as subsequent face-to-face follow-up requesting could be associated with the underlying diagnosis or management independent of technical factors. However, avoiding the diagnosis and speciality-specific information in this and similar analyses is likely to make any findings generalizable to a wider patient population, and potentially useful for updating generic guidance regarding remote consultations issued by national bodies such as that issued by NHSE [20]. The NRS used in this study were designed for this study and have not been previously validated. However, as noted earlier, on a literature search, there were no previously published validated tools identified for this purpose; our analysis shows that the NRS seem to have performed well in differentiating between dichotomous variables, suggesting they are valid tools for such an enquiry.

While remote consultations have served the need of the hour well during the pandemic for an unselected case-mix of non-urgent rheumatology patients, there seems to be scope for further improvement in overall effectiveness (Table 2). We have shown that achieving a satisfactory management plan is likely to reduce conversion to a subsequent face-to-face appointment. Areas were identified for implementing telemedicine both in the pre-pandemic era and also during the pandemic [18, 20]. Facilitators for telemedicine identified during this pandemic include less travel time for patients, ease of use of the system and shorter waiting period for patients [18]. These and similar issues are likely to remain important in future national/global emergencies. Also, with growing emphasis on climate change issues and reducing unnecessary travel, improvements that help achieve a satisfactory management plan would improve confidence and uptake of remote consultations in routine healthcare as well.

## Conclusions

We found that video consultations had a higher proportion of younger patients suggesting a preference for this amongst the younger patients. Video consultations were found to have similar ratings of management plan compared to phone consultations. Technical problems were noted in both video and phone consultations. Although they were more frequent in video consultations, this did not seem to impact negatively on the management plan itself. There was an inverse relationship of technical problems with subsequent face-to-face appointment requests, but the significance of this finding and relevance to clinical contexts will need to be explored in further studies for validation. Of the consultation process variables, lower rating of management plan was found to be the strongest predictor and also an independent predictor of a subsequent face-to-face appointment. From our study, we can infer that any developments in technology and resolving technical problems should be aimed towards achieving an adequate management plan comparable to that of a face-to-face consultation.

## Supplementary Information

Below is the link to the electronic supplementary material.

Supplementary file1 (PDF 433 KB)

Supplementary file2 (PDF 439 KB)

Supplementary file3 (PDF 463 KB)

## Data Availability

The data underlying this article will be shared on reasonable request to the corresponding author.

## References

[1] Kipps S, Paul A, Vasireddy S (2021). Incidence of COVID-19 in patients with rheumatic disease: is prior health education more important than shielding advice during the pandemic?. Clin Rheumatol.

[2] Jiménez E, Fontán-Vela M, Valencia J, Fernandez-Jimenez I, Álvaro-Alonso EA, Izquierdo-García E (2020). Characteristics, complications and outcomes among 1549 patients hospitalised with COVID-19 in a secondary hospital in Madrid, Spain: a retrospective case series study. BMJ Open.

[3] Strangfeld A, Schäfer M, Gianfrancesco MA, Lawson-Tovey S, Liew JW, Ljung L (2021). Factors associated with COVID-19-related death in people with rheumatic diseases: results from the COVID-19 Global Rheumatology Alliance physician-reported registry. Ann Rheum Dis.

[4] NHS England (2020) Clinical guide for the management of rheumatology patients during the coronavirus pandemic. Publications approval reference: 001559. Published 16 March 2020. https://www.england.nhs.uk/coronavirus/wp-content/uploads/sites/52/2020/03/clinical-guide-rheumatology-patients-v1-19-march-2020.pdf. Accessed 04 Feb 2022

[5] National Institute for Health and Care Excellence (2020) COVID-19 rapid guideline: rheumatological autoimmune, inflammatory and metabolic bone disorders. NICE guideline [NG167] Published: 03 April 2020; Last updated: 31 March 2021. https://www.nice.org.uk/guidance/NG167. Accessed 04 Feb 2022 33439591

[6] Price E, MacPhie E, Kay L, Lanyon P, Griffiths B, Holroyd C (2020). Identifying rheumatic disease patients at high risk and requiring shielding during the COVID-19 pandemic. Clin Med (Lond).

[7] McDougall JA, Ferucci ED, Glover J, Fraenkel L (2017). Telerheumatology: a systematic review. Arthritis Care Res (Hoboken).

[8] McKendrick JG (1878). Note on the microphone and telephone in auscultation. Br Med J.

[9] Stroganoff W (1928). Standard of results in the treatment of eclampsia. An experiment in the treatment of eclampsia by telephone consultation. Proc R Soc Med.

[10] Hennell S, Spark E, Wood B, George E (2005). An evaluation of nurse-led rheumatology telephone clinics. Musculoskelet Care.

[11] Mani-Babu S, Rita Abdulkader R (2019). Implementing nurse telephone follow-up clinics for monitoring parenteral osteoporosis treatment—a service evaluation. Clin Med.

[12] Graham LE, McGimpsey S, Wright S, McClean G, Carser J, Stevenson M (2000). Could a low-cost audio-visual link be useful in rheumatology?. J Telemed Telecare.

[13] Hammersley V, Donaghy E, Parker R, McNeilly H, Atherton H, Bikker A, Campbell J, McKinstry B (2019). Comparing the content and quality of video, telephone, and face-to-face consultations: a non-randomised, quasi-experimental, exploratory study in UK primary care. Br J Gen Pract.

[14] Nune A, Iyengar KP, Ahmed A, Bilgrami S, Sapkota HR (2021). Impact of COVID-19 on rheumatology practice in the UK-a pan-regional rheumatology survey. Clin Rheumatol.

[15] Mann DM, Chen J, Chunara R, Testa PA, Nov O (2020). COVID-19 transforms health care through telemedicine: evidence from the field. J Am Med Inform Assoc.

[16] Howren A, Aviña-Zubieta JA, Rebić N, Dau H, Gastonguay L, Shojania K, Davidson E, De Vera MA (2020). Virtual rheumatology appointments during the COVID-19 pandemic: an international survey of perspectives of patients with rheumatic diseases. Clin Rheumatol.

[17] Leggett P, Graham L, Steele K, Gilliland A, Stevenson M, O'Reilly D, Wootton R, Taggart A (2001). Telerheumatology–diagnostic accuracy and acceptability to patient, specialist, and general practitioner. Br J Gen Pract.

[18] Bos WH, van Tubergen A, Vonkeman HE (2021). Telemedicine for patients with rheumatic and musculoskeletal diseases during the COVID-19 pandemic; a positive experience in the Netherlands. Rheumatol Int.

[19] Chan A, Suarez A, Kitchen J, Bradlow A (2021). Teleclinics in rheumatology introduced during the first lockdown phase of the COVID-19 pandemic of 2020. Future Healthc J.

[20] Kruse CS, Krowski N, Rodriguez B, Tran L, Vela J, Brooks M (2017). Telehealth and patient satisfaction: a systematic review and narrative analysis. BMJ Open.

[21] NHS England (2020) Clinical guide for the management of remote consultations and remote working in secondary care during the coronavirus pandemic. Publications approval reference: 001559. Published 27 March 2020; updated November 2020. https://scts.org/_userfiles/pages/files/covid/2020/04/NHS-Clinical-Guide-for-the-Management-of-Remote-Consultations-and-Remote-Working-in-Secondary-Care-During-the-Coronavirus-Pandemic-27th-March-2020.pdf. Accessed on 04 Feb 2022

