# Supplementary material for: Technical factors can impact on remote consultations in rheumatology: results from a service evaluation during the COVID-19 pandemic
Source: Rheumatol Int. 2022 Apr 11;42(6):999–1007. doi: 10.1007/s00296-022-05112-5 (PMC8995407; doi:10.1007/s00296-022-05112-5)
Supplement: Supplementary file 1 — Supplementary file1 (PDF 433 KB) [file 296_2022_5112_MOESM1_ESM.pdf]

Online Resource 1

Journal: Rheumatology International

Article title: Technical factors can impact on remote consultations in Rheumatology – results from a service evaluation during the COVID-19 pandemic

Sreekanth Vasireddy[1,2], Consultant Rheumatologist & Hon. Senior Lecturer

Surabhi Wig[1,2], Consultant Rheumatologist & Hon. Senior Lecturer

Michael Hannides[1], Junior Doctor

[1]Department of Rheumatology

Bolton One Health Centre

Bolton NHS FT

Bolton, UK

[2]School of Biological Sciences,

University of Manchester

Manchester, UK

Corresponding author:

Sreekanth Vasireddy

Email: sreekanth.vasireddy@boltonft.nhs.uk

Online Resource 1 - Numerical Rating Scales Used In Data Collection Form (with 0 and 10 endpoint definitions)

Effectiveness *compared to face to face appointments (**please circle**):*

1. Was time available adequate for the consultation?

0	1	2	3	4	5	6	7	8	9	10

Very insufficient time **Ideal length of time**

2. Was relevant history obtained?

0	1	2	3	4	5	6	7	8	9	10

No relevant history obtained **All relevant history obtained**

3. Were physical exam findings obtained? ***(Please also tick if patient self-reported only ☐)***

0	1	2	3	4	5	6	7	8	9	10

Not able to examine **All relevant examination completed**

4. Was the remote consultation ideal to achieve a management plan for this patient?

0	1	2	3	4	5	6	7	8	9	10

No management plan possible **Full management plan achieved**

5. How would you rate the overall quality of communication possible during this consultation?

0	1	2	3	4	5	6	7	8	9	10

No relevant communication possible **All relevant communication completed**
