# Supplementary material for: Technical factors can impact on remote consultations in rheumatology: results from a service evaluation during the COVID-19 pandemic
Source: Rheumatol Int. 2022 Apr 11;42(6):999–1007. doi: 10.1007/s00296-022-05112-5 (PMC8995407; doi:10.1007/s00296-022-05112-5)
Supplement: Supplementary file 2 — Supplementary file2 (PDF 439 KB) [file 296_2022_5112_MOESM2_ESM.pdf]

Online Resource 2

Journal: Rheumatology International

Article title: Technical factors can impact on remote consultations in Rheumatology – results from a service evaluation during the COVID-19 pandemic

Sreekanth Vasireddy[1,2], Consultant Rheumatologist & Hon. Senior Lecturer

Surabhi Wig[1,2], Consultant Rheumatologist & Hon. Senior Lecturer

Michael Hannides[1], Junior Doctor

[1]Department of Rheumatology

Bolton One Health Centre

Bolton NHS FT

Bolton, UK

[2]School of Biological Sciences,

University of Manchester

Manchester, UK

Corresponding author:

Sreekanth Vasireddy

Email: sreekanth.vasireddy@boltonft.nhs.uk

Online Resource 2

Table 1 of 2. Types and frequencies of technical problems (one or more) recorded in the overall cohort (n=285)

Type	Frequency	Percent
Not recorded	26	9.1
No issues/problems	211	74.0
Other*	15	5.3
Other*; Poor Audio Quality	1	.4
Other*; Poor Audio Quality; Poor Connection; Poor Video Quality	1	.4
Other*; Poor Connection	2	.7
Poor Audio Quality	9	3.2
Poor Audio Quality; Poor Connection; Poor Video Quality	1	.4
Poor Audio Quality; Poor Video Quality	1	.4
Poor Audio Quality; User Capability	1	.4
Poor Connection	12	4.2
Poor Connection; Poor Video Quality	1	.4
User Capability	4	1.4
Total	285	100.0

*”Other” – descriptions in Table 2 of 2 below

Table 2 of 2. Description of technical problems coded in Table 1 of 2 as “Other” (individually or combined)

		Frequency	Percent
	Not coded as “other”	261	91.6
	Clinician had no access to laptop for video	1	.4
	Clinician had to switch laptop as no access initially	1	.4
	Delay	1	.4
	Did not log in	1	.4
	Failed video	1	.4
	Hearing aids	1	.4
	Hearing difficulties of patient	1	.4
	Limited views as mobile camera. Unable to assess joints very well	1	.4
	Line disconnected	1	.4
	No video available	1	.4
	Not able to log in for video call	1	.4
	On two extensions to patient and family member in different rooms	1	.4
	Patient called back after 3 attempts	1	.4
	Patient deaf	1	.4
	Patient did not log on for video consult	1	.4
	Patient driving when called. Paused until parked.	1	.4
	Patient partially deaf	2	.7
	Poor signal, had to switch to landline	1	.4
	Switched to landline from mobile	1	.4
	Video failed	1	.4
	Video froze	1	.4
	Was for video but connection failed	1	.4
	Wrong phone number, language barrier	1	.4
	Total	285	100.0
