# Supplementary material for: Technical factors can impact on remote consultations in rheumatology: results from a service evaluation during the COVID-19 pandemic
Source: Rheumatol Int. 2022 Apr 11;42(6):999–1007. doi: 10.1007/s00296-022-05112-5 (PMC8995407; doi:10.1007/s00296-022-05112-5)
Supplement: Supplementary file 3 — Supplementary file3 (PDF 463 KB) [file 296_2022_5112_MOESM3_ESM.pdf]

Online Resource 3

Journal: Rheumatology International

Article title: Technical factors can impact on remote consultations in Rheumatology – results from a service evaluation during the COVID-19 pandemic

Sreekanth Vasireddy[1,2], Consultant Rheumatologist & Hon. Senior Lecturer

Surabhi Wig[1,2], Consultant Rheumatologist & Hon. Senior Lecturer

Michael Hannides[1], Junior Doctor

[1]Department of Rheumatology

Bolton One Health Centre

Bolton NHS FT

Bolton, UK

[2]School of Biological Sciences,

University of Manchester

Manchester, UK

Corresponding author:

Sreekanth Vasireddy

Email: sreekanth.vasireddy@boltonft.nhs.uk

Online Resource 3

Table 1 of 2. Mean, median, skewness and kurtosis for variables in the overall cohort (n=285)

Report

	Age	Time Adequate	Relevant History	Physical Exam	Management Plan	Communication Quality
Mean	59.2656	8.50	8.75	1.60	7.22	8.11
Median	61.9932	9.00	9.00	.00	8.00	9.00
Skewness	-.417	-2.172	-1.155	1.718	-.978	-.933
Kurtosis	-.767	7.042	2.795	1.473	.616	.119

Table 2 of 2. Tests for normality of distribution for variables in the overall cohort (n=285)

Tests of Normality

	Kolmogorov-Smirnov[a]			Shapiro-Wilk		
	Statistic	df	Sig.	Statistic	df	Sig.
Age	.084	238	.000	.958	238	.000
Time Adequate	.215	238	.000	.822	238	.000
Relevant History	.189	238	.000	.869	238	.000
Physical Exam	.416	238	.000	.582	238	.000
Management Plan	.172	238	.000	.907	238	.000
Communication Quality	.193	238	.000	.875	238	.000

a. Lilliefors Significance Correction
